# Supplementary material for: Reorganization of Metabolism during Cardiomyogenesis Implies Time-Specific Signaling Pathway Regulation
Source: Int J Mol Sci. 2021 Jan 29;22(3):1330. doi: 10.3390/ijms22031330 (PMC7869011; doi:10.3390/ijms22031330)
Supplement: Supplementary file 1 [file ijms-22-01330-s001.zip › sup-944436/ijms-944436-supplementary.pdf]

Reorganization of metabolism during cardiomyogenesis implies time-specific signaling pathway regulation

Barisón et al., 2020

Supplementary Material

Supplementary Figures

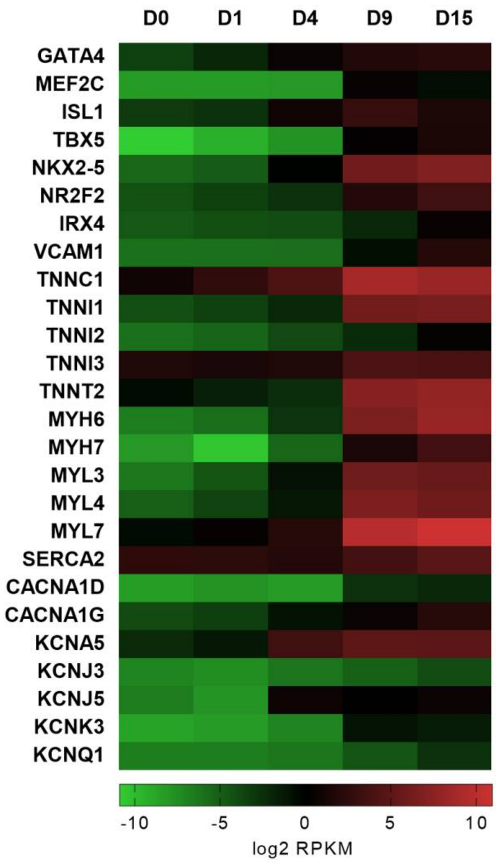

Figure S1: Expression pattern of cardiomyogenesis related genes. Heatmap of the expression levels of cardiac markers, such as cardiac regulators (GATA4, MEF2C, TBX5, ISL1, NKX2-5, NR2F2, IRX4, VCAM1), structural/sarcomeric genes (TNNC1, TNNI1, TNNI2, TNNI3, TNNT2, MYH6, MYH7, MYL3, MYL4, MYL7), calcium handling genes (SERCA2, CACNA1D, CACNA1G) and ion-channel genes (KCNA5, KCNJ3, KCNJ5, KCNK3, KCNQ1). As expected, the expression profile of these genes is in accordance with the cardiomyogenesis progression, presenting higher expression levels at cardiac progenitor (D9) and cardiomyocyte (D15) stages.

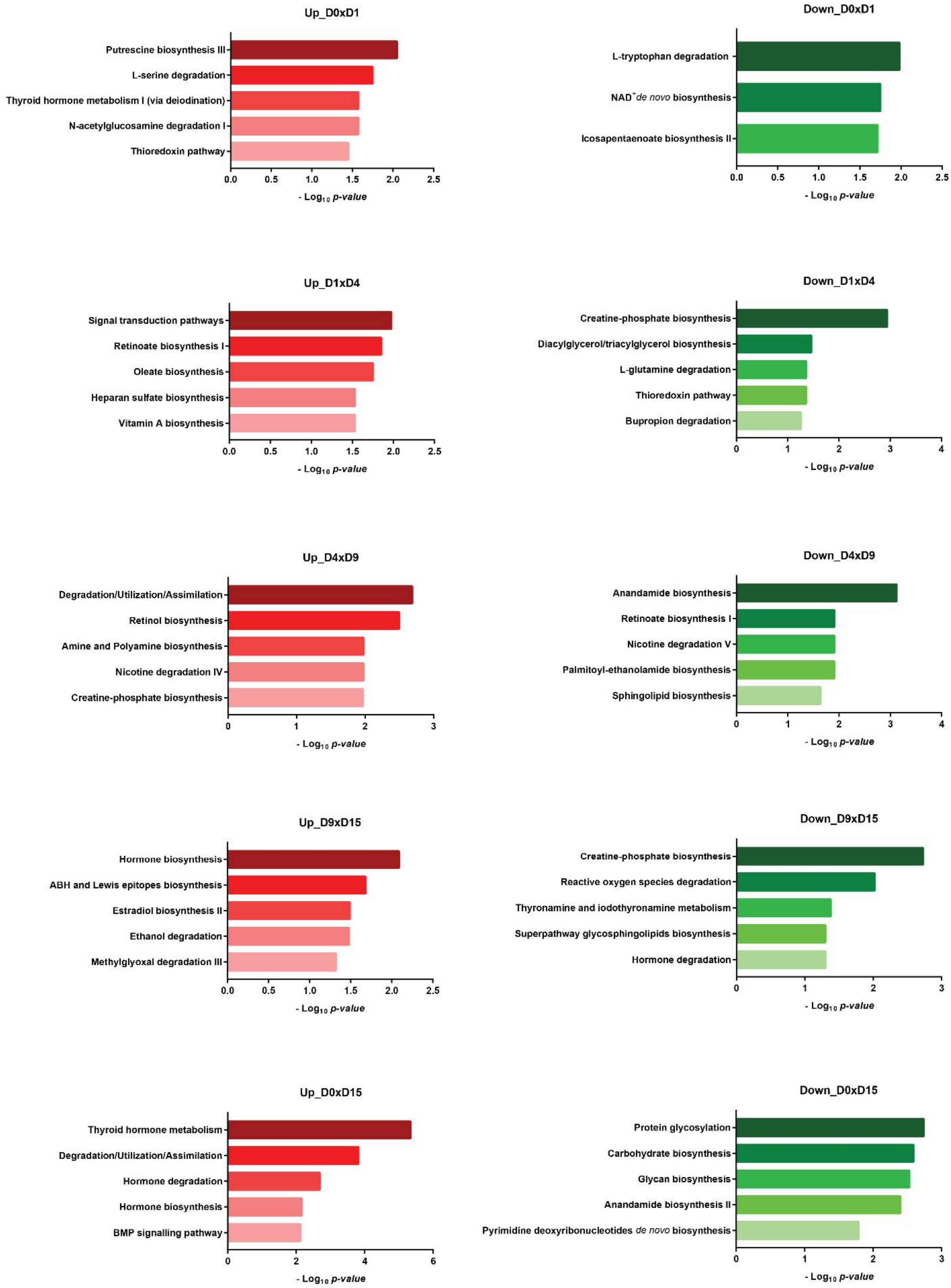

Figure S2: Metabolic pathways up- and downregulated during cardiomyogenesis according to BioCyc analysis. Enriched terms related to metabolism at each *in vitro* cardiac differentiation transition.

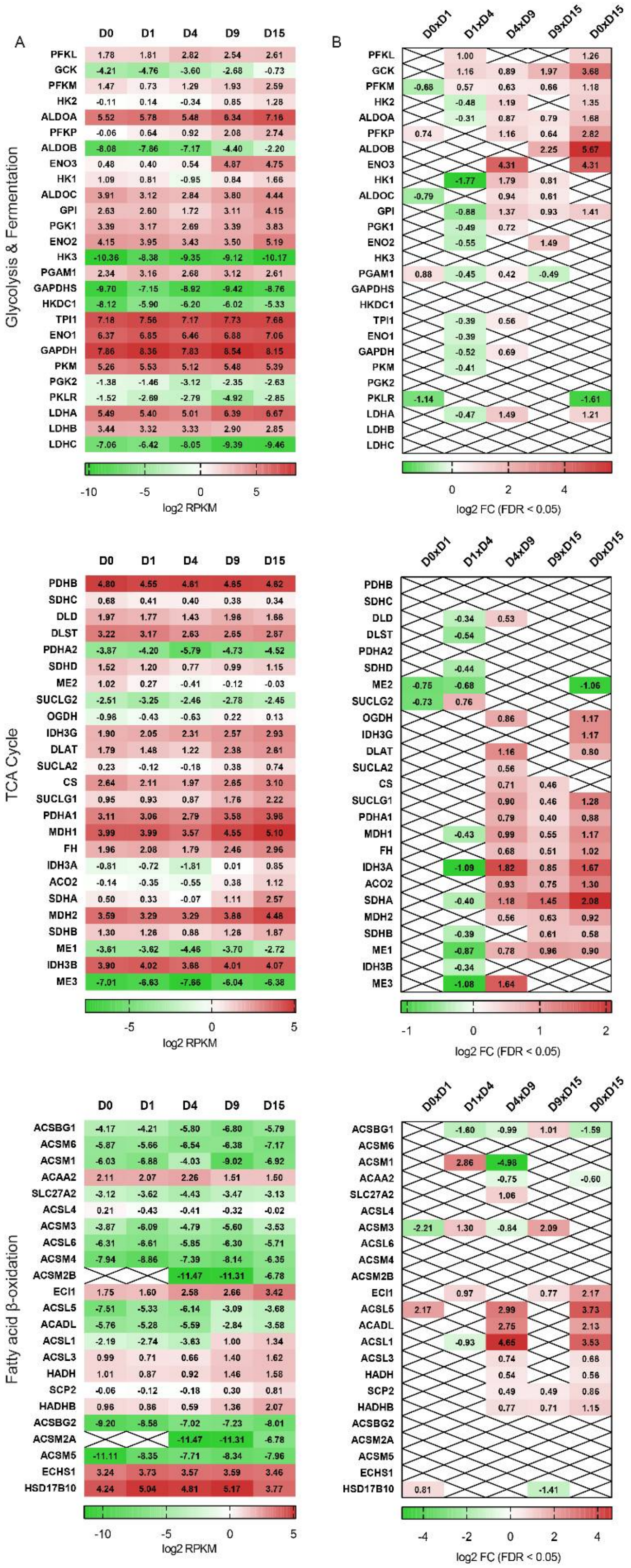

Figure S3: Energy metabolism-related pathways. Heatmaps representing the gene expression profiles (log2 RPKM values; **(A)**) and fold changes (log2 FC, FDR<0.05; **(B)**) of genes associated with glycolysis, fermentation, the TCA cycle and fatty acid β-oxidation. Red boxes indicate upregulated genes, while green boxes indicate downregulated genes.

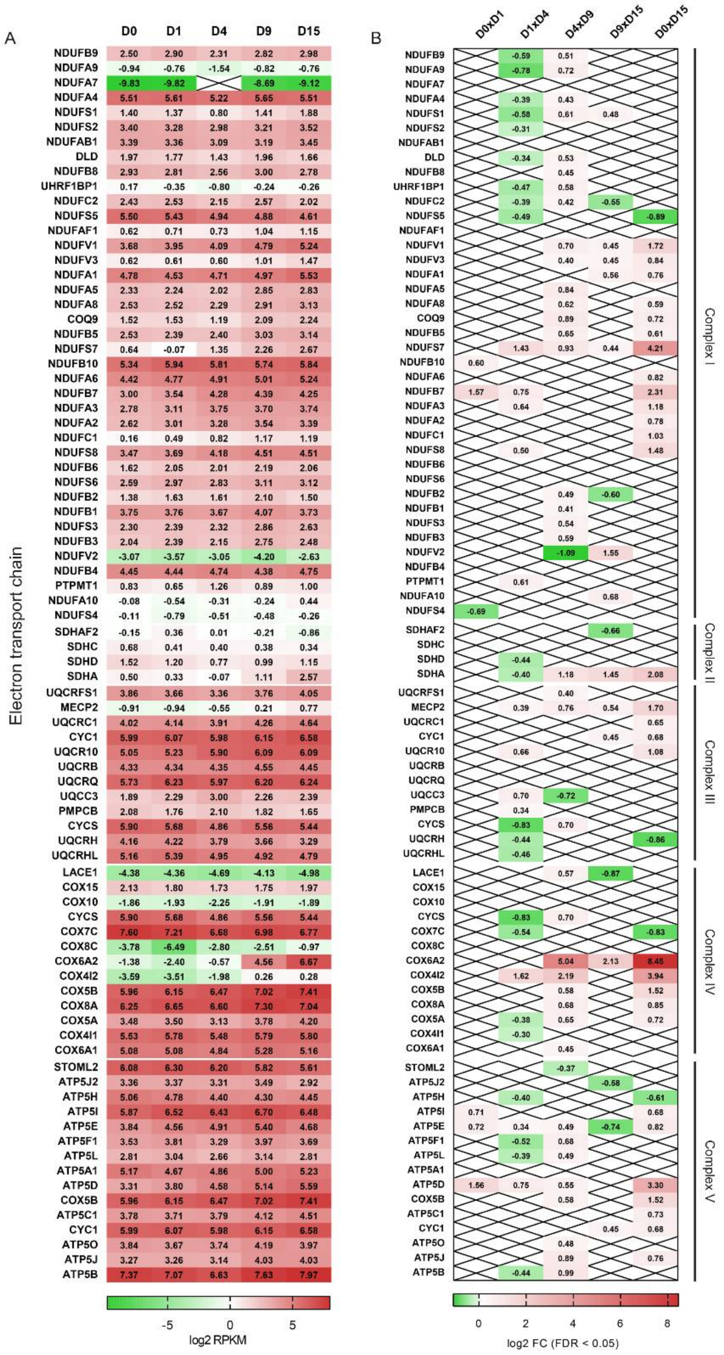

Figure S4: Energy metabolism-related pathways: electron transport chain (ETC). Heatmaps representing the gene expression profiles (log2 RPKM values; **(A)**) and fold changes (log2 FC, FDR<0.05; **(B)**) of genes encoding the five complexes of the ETC: complex I (NADH ubiquinone oxidoreductase), complex II (succinate dehydrogenase), complex III (cytochrome *bc*$_1$), complex IV (cytochrome *c* oxidase) and complex V (ATP synthase). Red boxes indicate upregulated genes, while green boxes indicate downregulated genes.

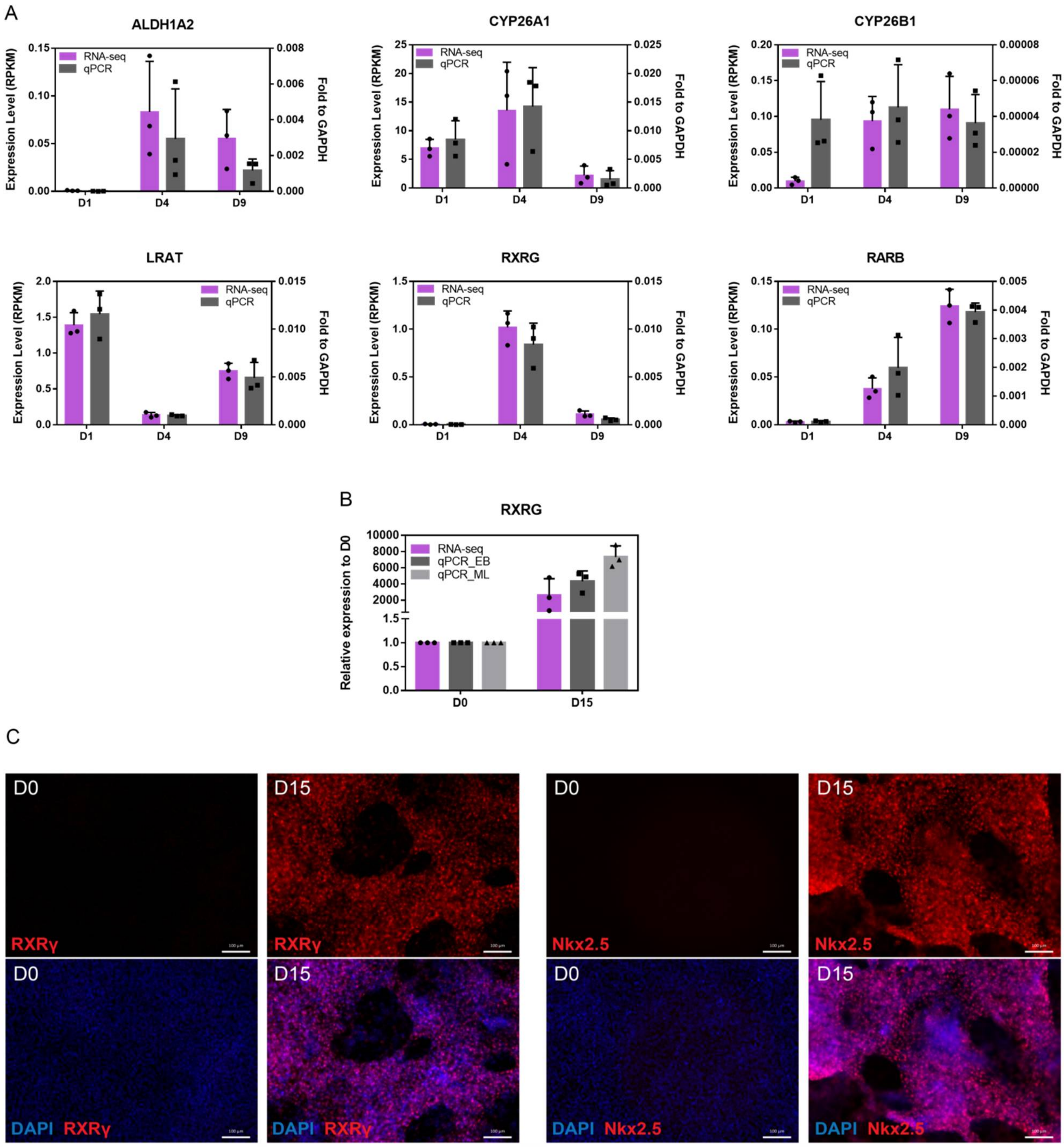

Figure S5: Transcript and protein expression validation for RA metabolism-related genes. (A) Differentially expressed genes obtained from RNA-seq data at D1, D4 and D9 stages were validated by qPCR. Expression level (RPKM) of each gene is compared to its relative expression to GAPDH, obtained by qPCR from polysome-bound RNA. The same expression pattern between RNA-seq and qPCR experiments was obtained for all evaluated genes. **(B)** Relative expression of RXRG was also validated by qPCR between D0 and D15, using embryoid body (EB) and monolayer (ML) differentiation protocols which corroborated with the expression pattern observed by RNA-seq. **(C)** RXRG expression at D0 and D15 was also validated by RXRγ protein expression by immunofluorescence assay. Left panel: RXRγ expression at D0 and D15. Right panel: Nkx2.5 expression at D0 and D15 was used as a positive cardiac differentiation marker. Cell nucleus was labeled using DAPI. Scale bar 100 μm.

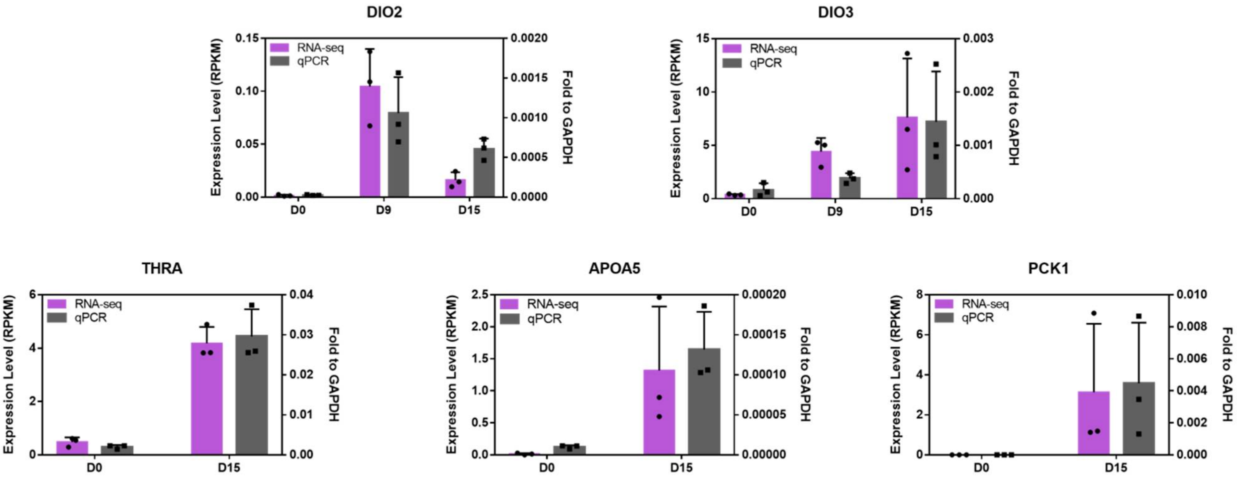

Figure S6: Transcript expression validation for TH metabolism-related genes. Differentially expressed genes obtained by RNA-seq experiments at specific times were validated by qPCR. Expression level (RPKM) of each gene is compared to its relative expression to GAPDH, obtained by qPCR. The same expression pattern between RNA-seq and qPCR experiments was obtained for all evaluated genes.

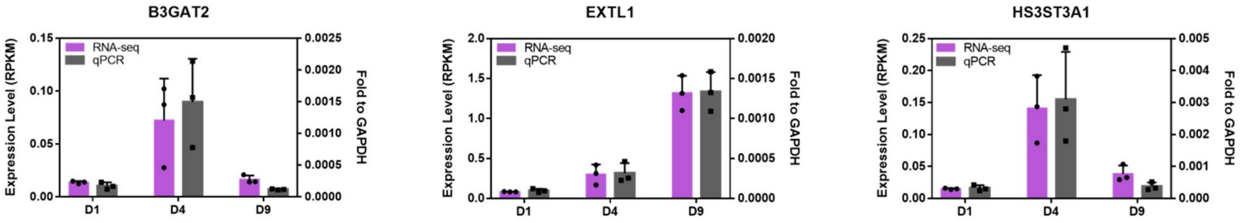

Figure S7: Transcript expression validation for heparan sulfate biosynthesis-related genes. Differentially expressed genes obtained by RNA-seq experiments at D1, D4 and D9 were validated by qPCR. Expression level (RPKM) of each gene is compared to its relative expression to GAPDH, obtained by qPCR. The same expression pattern between RNA-seq and qPCR experiments was obtained for all evaluated genes.

Supplementary Material and Methods

Monolayer cardiomyocyte differentiation and immunofluorescence assay

H1 hESCs were submitted to a protocol based on [1], with a few modifications. Briefly, 5 x 10^5 cells/well were seeded in Matrigel-coated 12-well plate in mTeSR-1 + 10 μM Y27632. After three days of daily medium change, differentiation was initiated with the addition of 12 μM CHIR99021 in RPMI/B-27 minus insulin medium (day 0) for 24h. On day 3, 10 μM XAV939 was added in RPMI/B-27 minus insulin medium for 48h. Besides that mentioned, RPMI/B-27 minus insulin medium was replaced every 2 days. From day 7 to day 15, the medium was replaced with RPMI/B-27 medium every 3 days. Beating areas were expected around day 10.

For immunofluorescence assays, at the indicated time-points, cells were fixed with 4% paraformaldehyde, permeabilized with 0.3% Triton X-100 for 30 min and blocked with 3% PBS-BSA for 1h. Then, it was performed an overnight incubation with anti-RXRγ or anti-NKX2-5 antibody (1:100 in 3% PBS-BSA) followed by incubation with Alexa 546-conjugated IgG secondary antibody (1:1000 in 3% PBS-BSA) and 1 μg/μL DAPI. Images were acquired using a Leica DMI6000B optical microscope.

Quantitative real time PCR

From EB cardiomyocyte differentiation protocol, polysome-bound RNA was isolated at the indicated time-points. From monolayer cardiomyocyte differentiation protocol, total RNA was isolated at D0 and D15 of differentiation. cDNA synthesis was performed using 1 μg of RNA and ImProm II Reverse Transcription System (Promega). Quantitative PCR (qPCR) was performed using GoTaq Polymerase Kit (Promega), primer sequences described in Table S9 and LightCycler 96 instrument and software (Roche). Each gene was assayed in three independent differentiations and qPCR technical triplicates each. Expression levels were calculated using the formula $2^{(-\Delta Ct)}$ and showed as "fold to GAPDH" or using the formula $2^{(-\Delta\Delta Ct)}$ and showed as "relative expression to D0", when indicated.

Table S9: Primer sequences used for expression validation by qPCR

Gene	Forward sequence (5′- 3′)	Reverse sequence (5′- 3′)	Amplicon size (bp)	Ref.
**Retinoic acid metabolism-related genes**				
ALDH1A2	GATGCTGACTTGGACTATGCTGT	CTGTTTCTTATCAATCTGGGGAC	210	[2]
CYP26A1	TCACTTACCTGGGGCTCTACC	ACTTGTTGTCTTGATTGCTCTTGC	96	[3]
CYP26B1	CCGCTTCCATTACCTCCCGTTC	CCACCGCCAGCACCTTCAG	89	[3]
LRAT	TGGAACAACTGCGAGCACTTCGTG	GCAGGAAGGGTAGTGTATGATACC	185	[4]
RXRG	CCGGATCTCTGGTTAAACACATC	GTCCTTCCTTATCGTCCTCTTGA	119	[5]
RARB	CTCACAGAGAAGATCCGAAAAGC	AATGCACTTGGTGGCCAGTT	150	[6]
**Thyroid hormone metabolism-related genes**				
DIO2	TCCAGTGTGGTGCATGTCTC	CTGGCTCGTGAAAGGAGGTC	168	[5]
DIO3	CTCTCCCTACATCATCCCACA	TGACATAGAGACGCTCGAAGTA	158	[5]
THRA	GCCTTTAACCTGGATGACACG	GTGTTTGCGGTGGTTGACG	153	[7]
APOA5	AAACCCTCTGGGGAGCATAC	CTAGGCTTCAACTTGGGAACC	75	[8]
PCK1	AGGATCGAAAGCAAGACGGT	GACGTACATGGTGCGACCTTT	165	--
**Heparan sulfate biosynthesis-related genes**				
B3GAT2	GCTGACGACGACAACACCTA	ACAACTTTGCCGTTTTCCAC	134	[9]
EXTL1	GGTCGTGTCGTGTCCTTTTC	GTTCTGTCTCTCCGCTCACC	136	[9]
HS3ST3A1	AAAATACAAGGCCCTGATAAAATTGA	CACTATGAAAACTGTGACGGGTACAA	130	[10]
**Normalizer gene**				
GAPDH	GGCGATGCTGGCGCTGAGTAC	TGGTTCACACCCATGACGA	149	--

Supplementary References

1. Lian, X.; Zhang, J.; Azarin, S.M.; Zhu, K.; Hazeltine, L.B.; Bao, X.; Hsiao, C.; Kamp, T.J.; Palecek, S.P. Directed cardiomyocyte differentiation from human pluripotent stem cells by modulating Wnt/β-catenin signaling under fully defined conditions. *Nat. Protoc.* **2013**, *8*, 162–175, doi:10.1038/nprot.2012.150.

2. Wang, Y.; Shao, F.; Chen, L. ALDH1A2 suppresses epithelial ovarian cancer cell proliferation and migration by downregulating STAT3. *Onco. Targets. Ther.* **2018**, *Volume 11*, 599–608, doi:10.2147/OTT.S145864.

3. Pavez Loriè, E.; Cools, M.; Borgers, M.; Wouters, L.; Shroot, B.; Hagforsen, E.; Törmä, H.; Vahlquist, A. Topical treatment with CYP26 inhibitor talarozole (R115866) dose dependently alters the expression of retinoid-regulated genes in normal human epidermis. *Br. J. Dermatol.* **2009**, *160*, 26–36, doi:10.1111/j.1365-2133.2008.08895.x.

4. Tang, X.H.; Suh, M.J.; Li, R.; Gudas, L.J. Cell proliferation inhibition and alterations in retinol esterification induced by phytanic acid and docosahexaenoic acid. *J. Lipid Res.* **2007**, *48*, 165–176, doi:10.1194/jlr.M600419-JLR200.

5. de Souza, J.S.; Ferreira, D.R.; Herai, R.; Carromeu, C.; Torres, L.B.; Araujo, B.H.S.; Cugola, F.; Maciel, R.M.B.; Muotri, A.R.; Giannocco, G. Altered Gene Expression of Thyroid Hormone Transporters and Deiodinases in iPS MeCP2-Knockout Cells-Derived Neurons. *Mol. Neurobiol.* **2019**, *56*, 8277–8295, doi:10.1007/s12035-019-01645-2.

6. Gutiérrez, J.; García-Villa, E.; Ocadiz-Delgado, R.; Cortés-Malagón, E.M.; Vázquez, J.; Roman-Rosales, A.; Alvarez-Rios, E.; Celik, H.; Romano, M.C.; Üren, A.; et al. Human papillomavirus type 16 E7 oncoprotein upregulates the retinoic acid receptor-beta expression in cervical cancer cell lines and K14E7 transgenic mice. *Mol. Cell. Biochem.* **2015**, *408*, 261–272, doi:10.1007/s11010-015-2504-1.

7. Poplawski, P.; Nauman, A. Thyroid hormone - triiodothyronine - has contrary effect on proliferation of human proximal tubules cell line (HK2) and renal cancer cell lines (Caki-2, Caki-1) - role of E2F4, E2F5 and p107, p130. *Thyroid Res.* **2008**, *1*, 5, doi:10.1186/1756-6614-1-5.

8. Pan, J.; Tong, S.; Tang, J. LncRNA expression profiles in HBV-transformed human hepatocellular carcinoma cells treated with a novel inhibitor of human La protein. *J. Viral Hepat.* **2018**, *25*, 391–400, doi:10.1111/jvh.12821.

9. Götte, M.; Spillmann, D.; Yip, G.W.; Versteeg, E.; Echtermeyer, F.G.; Van kuppevelt, T.H.; Kiesel, L. Changes in heparan sulfate are associated with delayed wound repair, altered cell migration, adhesion and contractility in the galactosyltransferase I (β4GalT-7) deficient form of Ehlers-Danlos syndrome. *Hum. Mol. Genet.* **2008**, *17*, 996–1009, doi:10.1093/hmg/ddm372.

10. Krenn, E.C.; Wille, I.; Gesslbauer, B.; Poteser, M.; van Kuppevelt, T.H.; Kungl, A.J. Glycanogenomics: A qPCR-approach to investigate biological glycan function. *Biochem. Biophys. Res. Commun.* **2008**, *375*, 297–302, doi:10.1016/j.bbrc.2008.07.144.
